# Real-Time PCR Quantification of Heteroplasmy in a Mouse Model with Mitochondrial DNA of C57BL/6 and NZB/BINJ Strains

**DOI:** 10.1371/journal.pone.0133650

**Published:** 2015-08-14

**Authors:** Thiago Simões Machado, Carolina Habermann Macabelli, Juliano Rodrigues Sangalli, Thiago Bittencourt Rodrigues, Lawrence Charles Smith, Flávio Vieira Meirelles, Marcos Roberto Chiaratti

**Affiliations:** 1 Departamento de Genética e Evolução, Centro de Ciências Biológicas e da Saúde, Universidade Federal de São Carlos, São Carlos, SP, 13565–905, Brazil; 2 Departamento de Cirurgia, Faculdade de Medicina Veterinária e Zootecnia, Universidade de São Paulo, São Paulo, SP, 05508–270, Brazil; 3 Departamento de Medicina Veterinária, Faculdade de Zootecnia e Engenharia de Alimentos, Universidade de São Paulo, Pirassununga, SP, 13635–900, Brazil; 4 Centre de recherche en reproduction animale, Faculté de Medecine Vétérinaire, Université de Montréal, Saint Hyacinthe, QC, J2S 7C6, Canada; Boston University School of Medicine, UNITED STATES

## Abstract

Mouse models are widely employed to study mitochondrial inheritance, which have implications to several human diseases caused by mutations in the mitochondrial genome (mtDNA). These mouse models take advantage of polymorphisms between the mtDNA of the NZB/BINJ and the mtDNA of common inbred laboratory (i.e., C57BL/6) strains to generate mice with two mtDNA haplotypes (heteroplasmy). Based on PCR followed by restriction fragment length polymorphism (PCR-RFLP), these studies determine the level of heteroplasmy across generations and in different cell types aiming to understand the mechanisms underlying mitochondrial inheritance. However, PCR-RFLP is a time-consuming method of low sensitivity and accuracy that dependents on the use of restriction enzyme digestions. A more robust method to measure heteroplasmy has been provided by the use of real-time quantitative PCR (qPCR) based on allelic refractory mutation detection system (ARMS-qPCR). Herein, we report an ARMS-qPCR assay for quantification of heteroplasmy using heteroplasmic mice with mtDNA of NZB/BINJ and C57BL/6 origin. Heteroplasmy and mtDNA copy number were estimated in germline and somatic tissues, providing evidence of the reliability of the approach. Furthermore, it enabled single-step quantification of heteroplasmy, with sensitivity to detect as low as 0.1% of either NZB/BINJ or C57BL/6 mtDNA. These findings are relevant as the ARMS-qPCR assay reported here is fully compatible with similar heteroplasmic mouse models used to study mitochondrial inheritance in mammals.

## Introduction

In mammals, the mitochondrial genome (mtDNA) encodes 13 polypeptides that are essential subunits of the enzyme complexes in the oxidative phosphorylation pathway [1,2]. As there are hundreds to several thousands of mtDNA molecules per cell, mutations in mtDNA are often present in a heteroplasmic state (i.e., coexistence of wild-type and mutant mtDNA) within single cells. Furthermore, due to poorly understood mechanisms, the mutation load varies markedly from one generation to another, which may result in mitochondrial dysfunction [1–4]. Since mutations in mtDNA have been implicated in several maternally-inherited human diseases [2], there is a growing interest in developing animal models to address the mechanisms underlying mitochondrial inheritance. For instance, several groups have developed heteroplasmic mice containing mtDNA of two different strains [5–13]. As the mtDNA haplotype of the NZB/BINJ (NZB) strain differs by dozens of nucleotides from the mtDNA haplotype of most laboratory inbred strains [8,14,15], including BALB/cByJ and C57BL/6 (B6), NZB mice are often used as a source of polymorphic mtDNA [5–12]. Hence, heteroplasmic mice are generated by mixing cytoplasm from two zygotes that differ in their mtDNA haplotype (i.e., NZB and B6 strains), followed by embryo transfer to foster mothers [6,7,11]. Since heteroplasmic mice are viable, the level of NZB mtDNA can be tracked down to study mitochondrial inheritance in different cell types and across generations [5–13].

The level of NZB mtDNA is estimated from heteroplasmic mice by comparing the amount of NZB mtDNA in relation to total mtDNA. This is most frequently performed by PCR amplification of a mtDNA fragment encompassing a polymorphic site present in one strain, but absent in the other [5,6,9,11,16–18]. Then, based on restriction fragment length polymorphism (RFPL) analysis it is possible to discriminate between mtDNA haplotypes using an endonuclease that cuts the PCR product from one haplotype but not from the other. Although widely employed, this method (PCR-RFLP) is time consuming and the use of radioactive material in last-cycle hot PCR-RFLP [18] engenders both risk- and cost-related disadvantages [19–22].

Alternatively, heteroplasmy may be determined by real-time quantitative PCR (qPCR) based on allelic refractory mutation detection system (ARMS), as reported for point mutations present in the human mtDNA (i.e., m.3243A>G, m.8993T>G and m.8993T>C) and models of mitochondrial inheritance [13,19–26]. ARMS-qPCR is based on the work by Newton et al. [27] in which mtDNA haplotypes differing by a single nucleotide polymorphism (SNP) can be selectively amplified by qPCR [13,19–24,26]. Discrimination in ARMS-qPCR is made by an oligonucleotide with the terminal 3’-nucleotide specific to one mtDNA haplotype. Thus, amplification of the non-target haplotype will be refractory because of the presence of a mismatched 3’-residue that prevents the oligonucleotide to function as primer. Furthermore, introduction of mismatched nucleotides immediately 5’ to the polymorphic site greatly increases oligonucleotide specificity allowing the quantification of heteroplasmic levels below 1% [19,20,22,24,26,27].

Herein we report the use of qPCR based on ARMS technology to quantify NZB mtDNA level from heteroplasmic cells. Using heteroplasmic mice and embryos we demonstrate the reliability of this approach in measuring heteroplasmy levels as low as 0.1%. Moreover, heteroplasmy and mtDNA copy number can be estimated from single pre-implantation embryos, without the need for preamplification. Therefore, researchers interested in investigating mitochondrial inheritance will benefit from this cost-effective approach, which enables a rapid, sensitive and accurate analysis of mtDNA haplotypes ratios in tissues and embryos from heteroplasmic mice.

## Material and Methods

All chemical and reagents used were purchased from Sigma-Aldrich Chemical Co. (St. Louis, MO, USA) unless otherwise stated. This study was carried out in strict accordance with the recommendations in the Guide for the Care and Use of Laboratory Animals of the National Institutes of Health and all efforts were made to minimize suffering. Mice were sacrificed by CO_2_ exposure and cervical dislocation. The protocol was approved by the Ethical Committee in the use of animals of the Faculdade de Zootecnia e Engenharia de Alimentos, Universidade de São Paulo (protocol number 13.1.1832.74.8).

### Source of mice and embryos

Mice containing mtDNA of NZB origin were obtained by mating founder pure NZB females to B6 males and backcrossing the female progeny to B6 males for five generations [6] and thereafter maintained by brother-sister mating. Due to elimination of sperm mtDNA after at fertilization (maternal inheritance), these mice contained exclusively NZB mtDNA haplotype under a 97% B6 nuclear genome. Mice containing mtDNA of B6 origin were obtained from F_1_ females from a cross between B6 females and males of the CBA strain. These females containing NZB or B6 mtDNA are hereafter termed NZB and B6, respectively. For zygote collection, females were superovulated by intra-peritoneal injection of 5 i.u. of equine chorionic gonadotropin (eCG; Folligon, MSD Animal Health, Summit, USA) and 5 i.u. of human chorionic gonadotropin (hCG; Chorullon, MSD Animal Health) given 46–47 h apart [28]. After hCG injection, females were paired with F_1_ males (derived from a cross between B6 females and CBA males) and inspected for the presence of a copulation plug at the following morning. Fertilized embryos were flushed at 18 h after hCG injection from the oviducts using HEPES-buffered KSOM medium (FHM) [28]. Zygotes were denuded of cumulus cells by pipetting in 0.3% hialuronidase solution, washed in FHM and cultured under mineral oil in groups of 20 embryos in 40-μl droplets of KSOM medium [28]. Embryos were cultured in humidified incubators at 37°C in air with 5% CO_2_. Blastocysts were obtained after 96 h of culture. Mature oocytes were collected similarly to that described to zygotes with exception that donor females were not paired with males.

### Production of heteroplasmic embryos and mice

Microsurgery was performed using an inverted microscope (Leica DMI RB; Leica, Wetzlar, Germany) equipped with micromanipulators and microinjectors (Narishige, Tokyo, Japan) based on the report by Ferreira et al [29], with few modifications. Briefly, pronuclear zygotes were placed under mineral oil in a 100-μl droplet of FHM supplemented with 5 μg/ml cytochalasin B and 0.5 μg/ml nocodazole [6]. Using a 15-μm (internal diameter) glass pipet (Eppendorf, Hamburg, Germany), a cytoplasmic biopsy of about 30% of the embryo’s volume was removed from NZB zygotes and subsequently introduced into the perivitelline space of B6 zygotes from which a similar cytoplasm volume had previously been removed. The resulting couplets were placed in electrofusion solution (0.28 M mannitol, 0.1 mM MgSO4, 0.5 mM HEPES and 0.05% BSA) and exposed to a single electrical pulse of 1 kV/cm for 45 μs (Multiporator, Eppendorf). Alternatively, NZB and B6 zygotes were incubated for 30 min in FHM supplemented with 5 μg/ml cytochalasin B and 0.5 μg/ml nocodazole prior to micromanipulation followed by centrifugation at 15.000 x g for 10 min. Zygote centrifugation leads to formation of a mitochondrial-enriched cytoplasmic fraction [30–32], enabling generation by cytoplasmic transfer of reconstructed zygotes with higher levels of heteroplasmy [29]. Thus, after centrifugation zygotes were subjected to the procedure of cytoplasmic transfer as described above but using the mitochondrial-enriched cytoplasmic fraction. Successfully fused zygotes were selected and cultured to the blastocyst stage as described above. Part of the reconstructed zygotes was transferred into the oviducts of Swiss females mated to vasectomized Swiss males [28]. Anesthesia was performed by intraperitoneal injection of 100 mg/kg Ketamine (Dopalen, Ceva Santé Animale, Paulínia, Brazil) and 16 mg/kg Xylazine (Rompun, Bayer, São Paulo, Brazil) [28]. Females derived from cytoplasmic transfers (founders) were mated to B6 males to obtain first generation progeny (BC_1_). Thereafter, female progeny from each generation (BC_1_, BC_2_, BC_3_ and BC_4_) were selected based on heteroplasmic levels and backcrossed (BC) with B6 males. This heteroplasmic mouse lineage was termed B6/NZB.

### Sampling and DNA preparation

Genomic DNA (gDNA) used in qPCR reactions was extracted from tissue biopsies (tail, ear, liver, heart or brain) using a standard protocol [11,33]. Briefly, samples were incubated at 55°C for 3 h in 500 μl of a solution containing 0.4 mM NaCl, 0.02 M Tris-Cl (pH 8.0), 5 mM EDTA (pH 8.0), 1% SDS and 0.4 mg/ml proteinase K. Protein was extracted using 25:24:1 phenol/chloroform/isoamyl alcohol and then 24:1 chloroform/isoamyl alcohol. DNA was precipitated using isopropyl alcohol, washed in 70% ethanol and eluted in ultrapure water. Samples were evaluated by UV spectroscopy at wavelengths of 260, 280 and 230 nm and aliquoted at concentration of 2.5 ng/μl of DNA.

Ear biopsies obtained using an ear punch were also prepared using an alternative protocol described by Bouma et al. [34]. According to this protocol a small biopsy of tissue (a circle of ~2 mm of diameter) was digested for 3 h at 55°C in 200 μl of a solution containing 50 mM KCl, 10 mM Tris-Cl (pH 8.3), 2 mM MgCl_2_, 0.1 mg/ml gelatin, 0.45% Igepal CA-630, 0.45% Tween 20 and 100 μg/ml proteinase K. After digestion, samples were incubated at 95°C for 10 min for inactivation of proteinase K. Tissue lysates were then centrifuged at 10,000 x g for 5 min and 0.5 μl of the supernatant was diluted 1:100 to be used in qPCR reactions.

With respect to oocytes and embryos, these were washed three times in filtered PBS containing 0.1% polyvinyl-pyrrolidone (PVP). They were then placed individually into 0.2-ml microtubes containing 1 μl of PBS plus 0.1% PVP and stored at -20°C until further use. For use in qPCR, oocytes and embryos were digested as described above for ear biopsies by adding 4 μl of the digestion solution. However, as reported by Shitara et al. [35], the digestion solution contained 100 mg/ml proteinase K. After proteinase K inactivation, the lysate was diluted by addition of 45 μl of ultrapure water, centrifuged at 10,000 x g for 5 min and the supernatant used for analysis of heteroplasmy and mtDNA copy number.

### Analysis of mitochondrial heteroplasmy by ARMS-qPCR

Mitochondrial heteroplasmy was measured by parallel amplification of NZB and B6 mtDNA in independent reactions by qPCR based on ARMS technology [13,19,20,22,24,26,27]. Therefore, mtDNA sequences available on Genbank for NZB (L07095.1) [15] and B6 (NC_005089.1) [14] strains were aligned allowing identification of 106 SNPs [8]. Based on this analysis and using the Primer Express software (Life Technologies, Carlsbad, USA) it was possible to design two primer pairs that amplify selectively either NZB or B6 mtDNAs (Table 1). Primers ARMS22 and MT20 were designed (S1 File) to amplify a 118-bp fragment encompassing nucleotides 3,578 to 3,695 of NZB mtDNA (part of *mt-Nd1* gene) whereas primers ARMS2 and MT14 amplify a 146-bp fragment encompassing nucleotides 3,815 to 3,960 of B6 mtDNA (part of *mt-Tq*, *mt-Tm* and *mt-Nd2* genes). qPCR reactions consisted of 15 μl containing 200 nM of each primer (ARMS22 and MT20 or ARMS2 and MT14), 1x Power SYBR Green Master Mix (Life Technologies) and 2 μl (somatic tissue) or 5 μl (oocytes or embryos) of DNA. Amplifications were performed using the 7500 Fast Real-Time PCR System (Life Technologies) with the following cycling conditions: initial denaturation at 95°C for 10 min followed by 40 cycles of 95°C for 15 sec and 62°C for 1 min. SYBR Green fluorescence was measured at the end of each extension step (62°C). Specificity of the amplified fragments was confirmed by melt-curve analysis and electrophoresis of the PCR products on 2% agarose gel. Samples were analyzed in duplicates or triplicates and averaged for calculation of heteroplasmy. Standard curves were generated by qPCR using as template 5-fold serial dilutions of heteroplasmic DNA (25, 5, 1 and 0.2 ng per reaction). As suggested by the manufacturer, a first-degree linear regression was fitted for the log of input amount of template versus the Ct (cycle threshold) and the delta (∆) Ct values for serial-diluted DNAs [36]. The ∆Ct was calculated by subtracting Ct_B6_ values from Ct_NZB_ values. Amplification efficiency for each primer pair was estimated based on the slope values from the linear regression (log of DNA vs. Ct values) as follows: efficiency = 10^^(-1/slope)^. On the other hand, amplification efficiency was compared between B6 and NZB assays based on the slope values from other linear regression (log of DNA vs. ∆Ct values). In the latter case, if the slope values are equal or smaller than 0.1, the level of NZB mtDNA can estimated in relation to the sum of NZB and B6 mtDNA [36].

**Table 1:** Primer sequences used for measuring heteroplasmy and mtDNA copy number.

Target	Primer	Sequence (5’-3’)^a^	Product (bp)
mtDNA^b^	MT12	CGCCCTAACAACTATTATCTTCC	736
	MT13	GACCGTTTGTTTGTTGTTGAAA	
mtDNA^b^	MT14	CTCCGTGCTACCTAAACACCTTATC	148
	MT15	GACCTAAGAAGATTGTGAAGTAGATGATG	
B6 mtDNA^c^	MT14	CTCCGTGCTACCTAAACACCTTATC	146
	ARMS2	CCTAAGAAGATTGTGAAGTAGATGATGtC	
NZB mtDNA^d^	MT20	TGGCACTCCCGCTGTAAAAA	118
	ARMS22	TTATCCACGCTTCCGTTACGtC	
*Apob* (nuclear DNA)	OIMR1544	CACGTGGGCTCCAGCATT	74
	OIMR3580	TCACCAGTCATTTCTGCCTTTG	

^a^Underlined nucleotides are complementary to one of mtDNA haplotypes (NZB or B6) due to the presence of a polymorphic nucleotide at position 3,599 (ARMS22) and 3,932 (ARMS2), respectively. A mismatched nucleotide introduced immediately 5’ to the polymorphic sites is represented in lowercase.

^b^Non-driscriminative assays that amplify mtDNA of B6 and NZB origin.

^c^Driscriminative assay that amplifies mtDNA of B6 origin.

^d^Driscriminative assay that amplifies mtDNA of NZB origin.

### Quantification of mtDNA copy number

The number of mtDNA copies was estimated in oocytes and whole embryos based on a previous report [9]. Briefly, using primers MT12 and MT13 a 736-bp fragment encompassing nucleotides 3,455 to 4,190 of both B6 and NZB mtDNAs was amplified and cloned into a pCR2.1-TopoTA (Life Technologies). Based on UV spectroscopy, the resulting plasmid DNA (pDNA) was aliquoted at concentration of 0.2 x 10^9^ copies/μl and kept at -80°C until use. For quantification of mtDNA copy number a standard curve was prepared by dilution of the pDNA to 10^7^, 10^6^, 10^5^, 10^4^ and 10^3^ copies per reaction. This standard curve was amplified in parallel with samples containing 10% of embryo lysate (5 μl from a total of 50 μl of lysate). Amplification was performed as described for quantification of heteroplasmy, but using primers MT14 and MT15 (Table 1). These non-discriminative primers amplify a 148-bp fragment encompassing nucleotides 3,815 to 3,962 of NZB and B6 mtDNA (part of *mt-Tq*, *mt-Tm* and *mt-Nd2* genes). Based on the standard curve values, it was possible to estimate mtDNA copy number in each sample using the 7500 Software (v. 2.0.6; Life Technologies).

The number of mtDNA copies per cell was determined in somatic tissues by normalization of mtDNA amount against a single copy nuclear gene (*Apob*) [37]. Primers MT14 and MT15 were used to amplify mtDNA whereas primers OIMR1544 and OIMR3580 were used to amplify a 74-bp fragment from *Apob* gene (Table 1). Both reactions were performed in parallel and following the amplification conditions described above. The amplification efficiency of mtDNA and *Apob* assays was analyzed as described above (see item 2.4.). The number of mtDNA copies per cell was then determined as reported by Nicklas et al. [37].

### Statistics

Statistical analyses were performed using the SAS System (v. 9.3; Cary, USA). All data were tested for assumption of normal distribution and homogeneity of variance. When needed, data were transformed (log or square root) to meet these criteria. Pearson’s correlation coefficient (r) was used for analysis of the relationship between two variables. The level of NZB mtDNA in embryos was analyzed by two-way ANOVA considering two developmental stages (zygote and blastocyst) and two experimental groups (centrifuged and non-centrifuged embryos). Remaining data were analyzed by one-way ANOVA, followed by Tukey’s post-hoc test. Differences with probabilities (P) < 0.05 were considered significant. Values are reported as mean ± the standard error of the mean (SEM). In some instances, the coefficient of variance (CV) is also reported.

## Results

### Reliability of the assay for quantification of heteroplasmy

**Fig 1:**
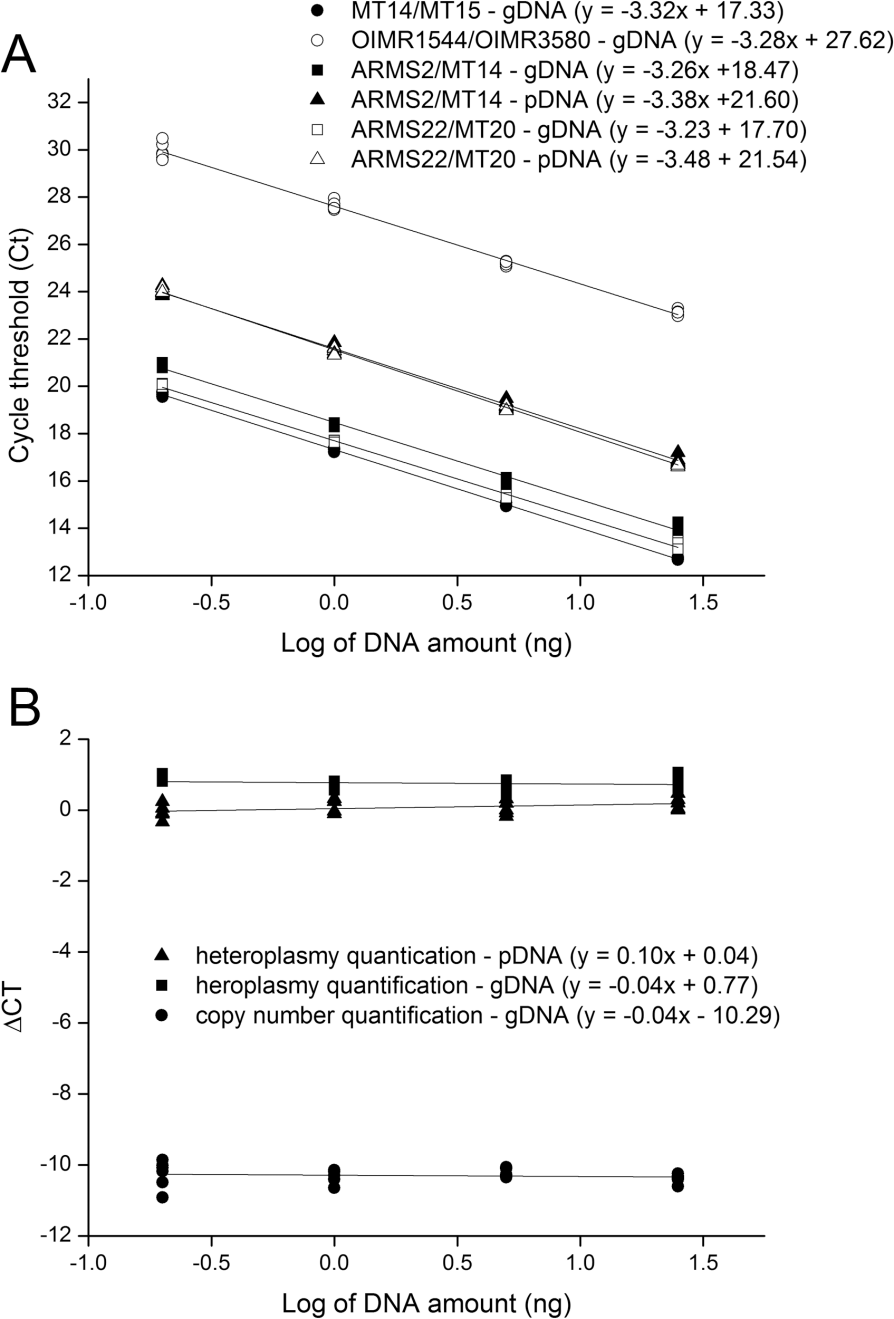
Analysis of primer efficiency. Standard curves were generated by qPCR using as template 5-fold serial dilutions of heteroplasmic gDNA or pDNA (25, 5, 1 and 0.2 ng per reaction). First-degree linear regressions were fitted for the log of input amount of template versus the Ct (A) or the ∆Ct (B) values for serial-diluted DNAs. The ∆Ct was calculated by subtracting either Ct_B6_ from Ct_NZB_ (heteroplasmy quantification) or Ct_mtDNA_ from Ct_Apob_ (copy number quantification). Comparison of amplification efficiency between primer pairs (ARMS2/MT14 vs. ARMS22/MT20 and MT14/MT15 vs. OIMR1544/OIMR3580) was found similar as the slope values from linear regressions were equal or smaller than 0.1 (B). Moreover, the use of gDNA or pDNA for heteroplasmy quantification did not affect slope values.

**Fig 2:**
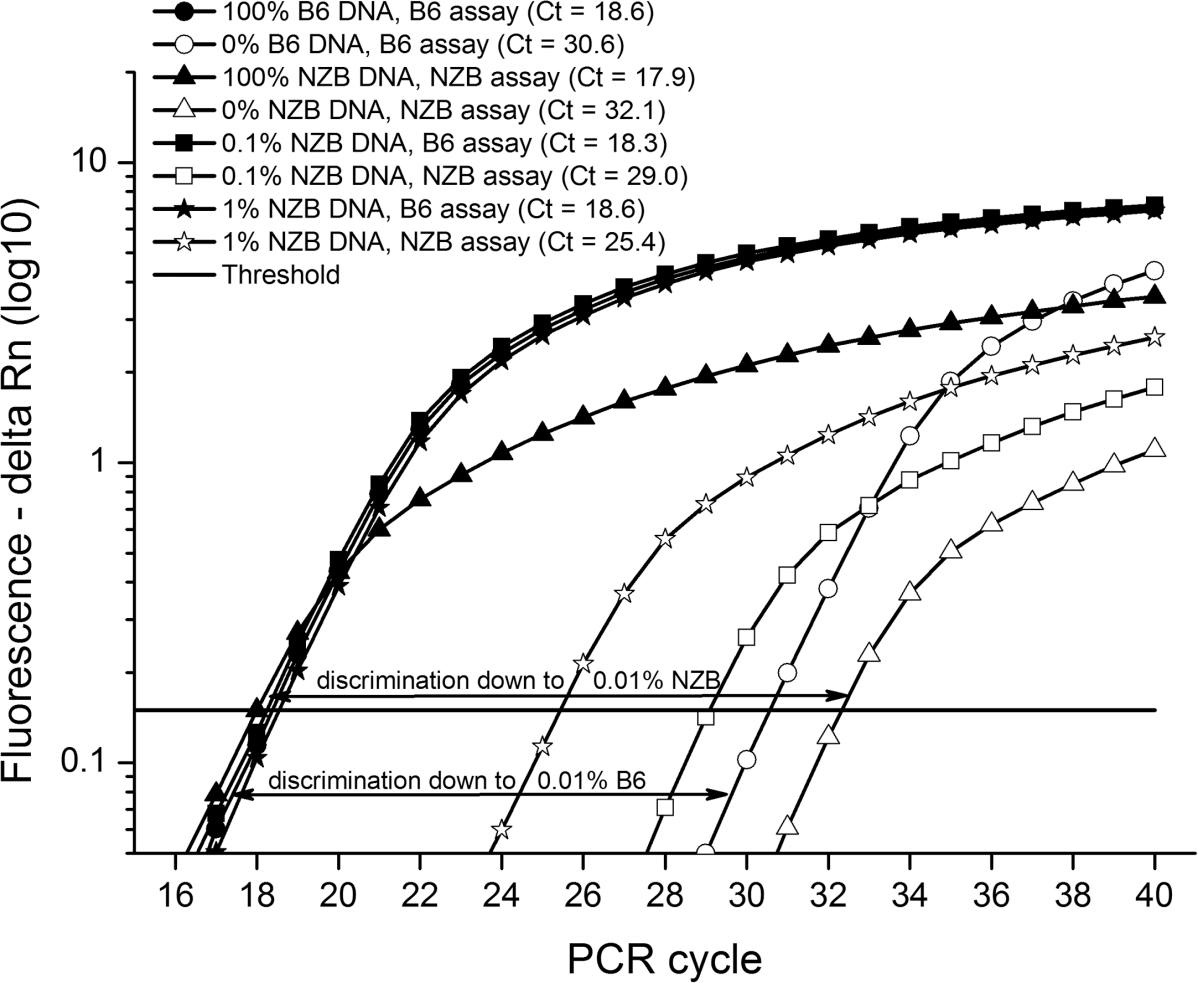
Specificity of the ARMS-qPCR approach for amplification of target mtDNA. Using samples from homoplasmic mice (either NZB or B6), non-specific amplification of non-target mtDNA accounted for only 0.01% of target amplification. Analysis of samples from heteroplasmic mice showed that levels as low as 0.1% of mtDNA of NZB or B6 origin could be detected. Individual Ct values (cycle at which plots crossed the threshold) are denoted in the inset.

**Fig 3:**
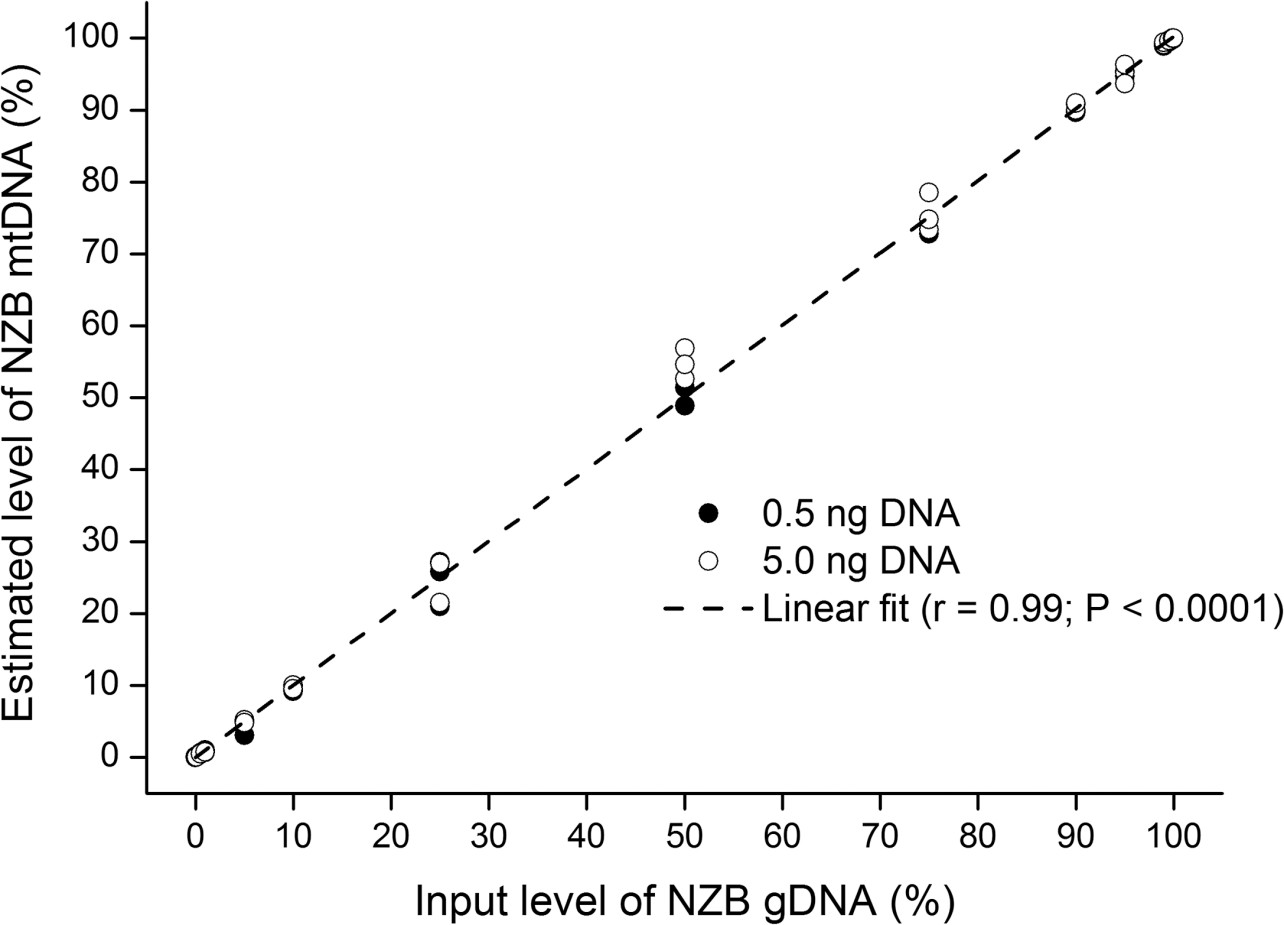
Relationship between the input level of NZB gDNA and the level of NZB mtDNA estimated by ARMS-qPCR. Different proportions of gDNA from NZB mice (100%, 99.9%, 99.5%, 99%, 95%, 90%, 75%, 50%, 25%, 10%, 5%, 1%, 0.5%, 0.1% and 0%) were mixed with gDNA from B6 mice to be analyzed by ARMS-qPCR. Filled circles depict the use of 0.5 ng of DNA per reaction (duplicates) whereas empty circles depict the use of 5.0 ng of DNA (triplicates). The relationship between the input level of NZB gDNA and the level of NZB mtDNA determined by ARMS-qPCR was analyzed by calculating Pearson’s correlation coefficient (r). P value is denoted in the inset.

At first, we sought to investigate whether primers designed based on ARMS technology could be used for quantification of heteroplasmy by qPCR. Comparison of the assays used for amplification of mtDNA of NZB and B6 origin (Table 1) showed that both primer pairs have high (>93%) and similar (slope ≤ 0.1) efficiency of amplification (Fig 1). Quantification of heteroplasmy using these primers also proved to be efficient, with sensitivity to detect as low as 0.1% of either NZB or B6 mtDNA (Fig 2). Non-specific amplification of non-target mtDNA (i.e., NZB mtDNA) accounted for only 0.01% of target amplification when a homoplasmic sample (i.e., B6 mtDNA) was used (Fig 2). Even when different proportions of gDNA from NZB and B6 homoplasmic mice were mixed, the assay was capable of estimating the percentage of NZB mtDNA with great efficiency (Fig 3). Regardless of the use of 5.0 or 0.5 ng of DNA per reaction, a very consistent relationship (r = 0.99; P < 0.0001) was found between the input level of NZB gDNA and the level of NZB mtDNA estimated by qPCR (Fig 3). Furthermore, the CV of the intra-run and inter-run were equal to 4.6% ± 1.33 and 2.7% ± 0.86, respectively, when comparing the level of NZB mtDNA among triplicates. In summary, these results gave evidence of the efficiency and sensitivity of the approach, which supports its use for measurement of NZB mtDNA level in heteroplasmic mouse samples.

### Analysis of heteroplasmy in germline and somatic tissues

**Fig 4:**
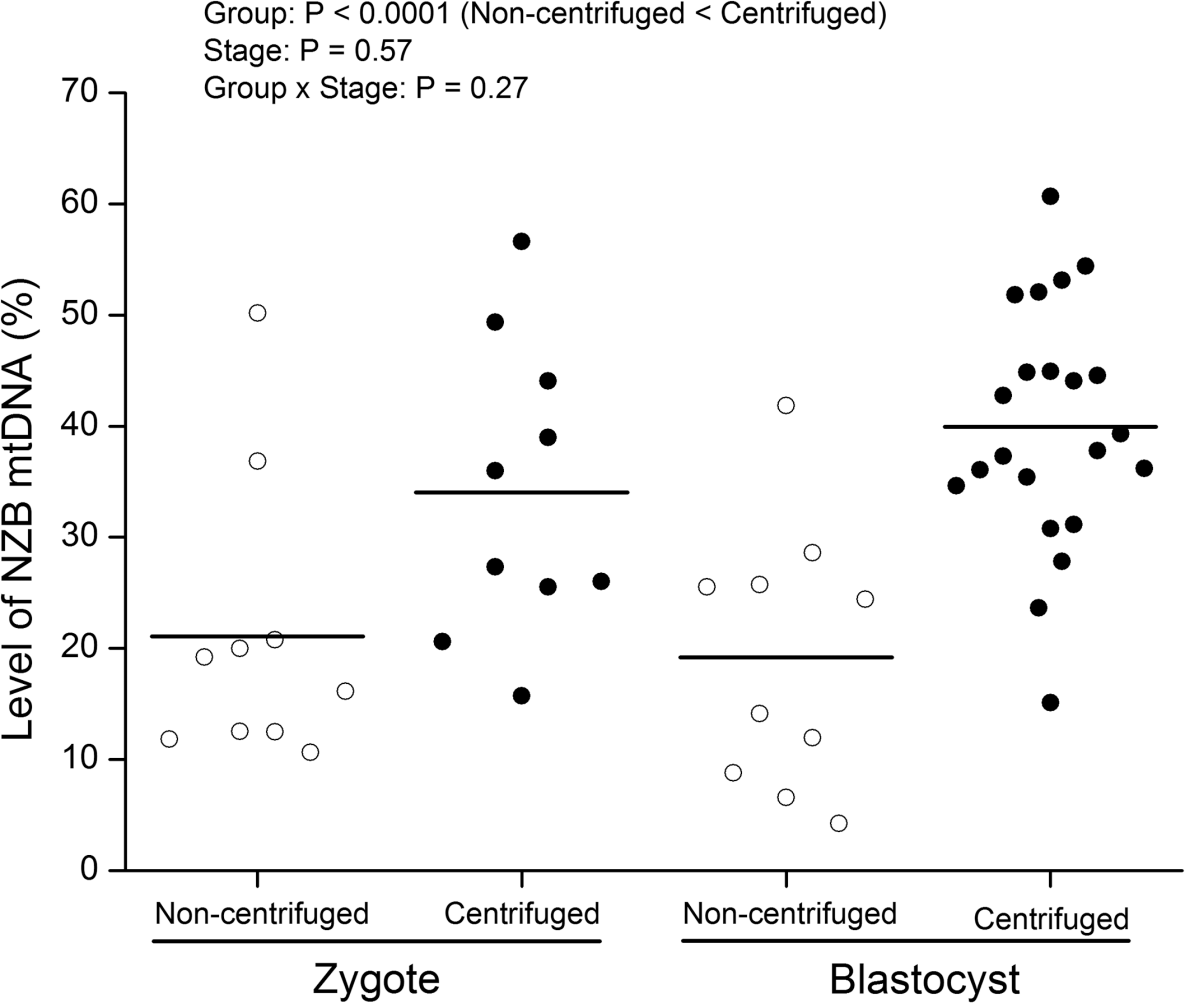
Level of NZB mtDNA in heteroplasmic mouse embryos produced by cytoplasmic transfer. Zygotes were centrifuged prior to cytoplasmic transfer to enable production of embryos with higher levels of NZB mtDNA (filled circles). For comparison, cytoplasmic transfer was also performed without centrifugation (empty circles). Embryos from both groups were evaluated with regards to the level of NZB mtDNA at the zygote and blastocyst stages by ARMS-qPCR. Bars represent the means. P values for the effect of Group, Stage and Group x Stage are denoted in the inset.

Next, we aimed at confirming the reliability of ARMS-qPCR assay by measuring the level of NZB mtDNA in embryos produced by two methods based on cytoplasmic transfer (Fig 4). According to our measurements, zygotes from the centrifuged group contained 34.0% ± 3.76 (range, 15.7–56.6) of NZB mtDNA in comparison to 21.1% ± 3.76 (range, 10.7–50.2) present in zygotes from the non-centrifuged group (Fig 4). Culture of heteroplasmic embryos to the blastocyst stage confirmed these findings (Fig 4) as blastocysts from the centrifuged group contained 39.9% ± 2.54 (range, 15.1–60.7) of NZB mtDNA whereas blastocysts from the non-centrifuged group contained 19.2% ± 3.76 (range, 4.3–41.9). Thus, consistent with our previous report [29], embryos from the centrifuged group contained, regardless of the developmental stage (P < 0.0001), more NZB mtDNA than non-centrifuged embryos.

**Fig 5:**
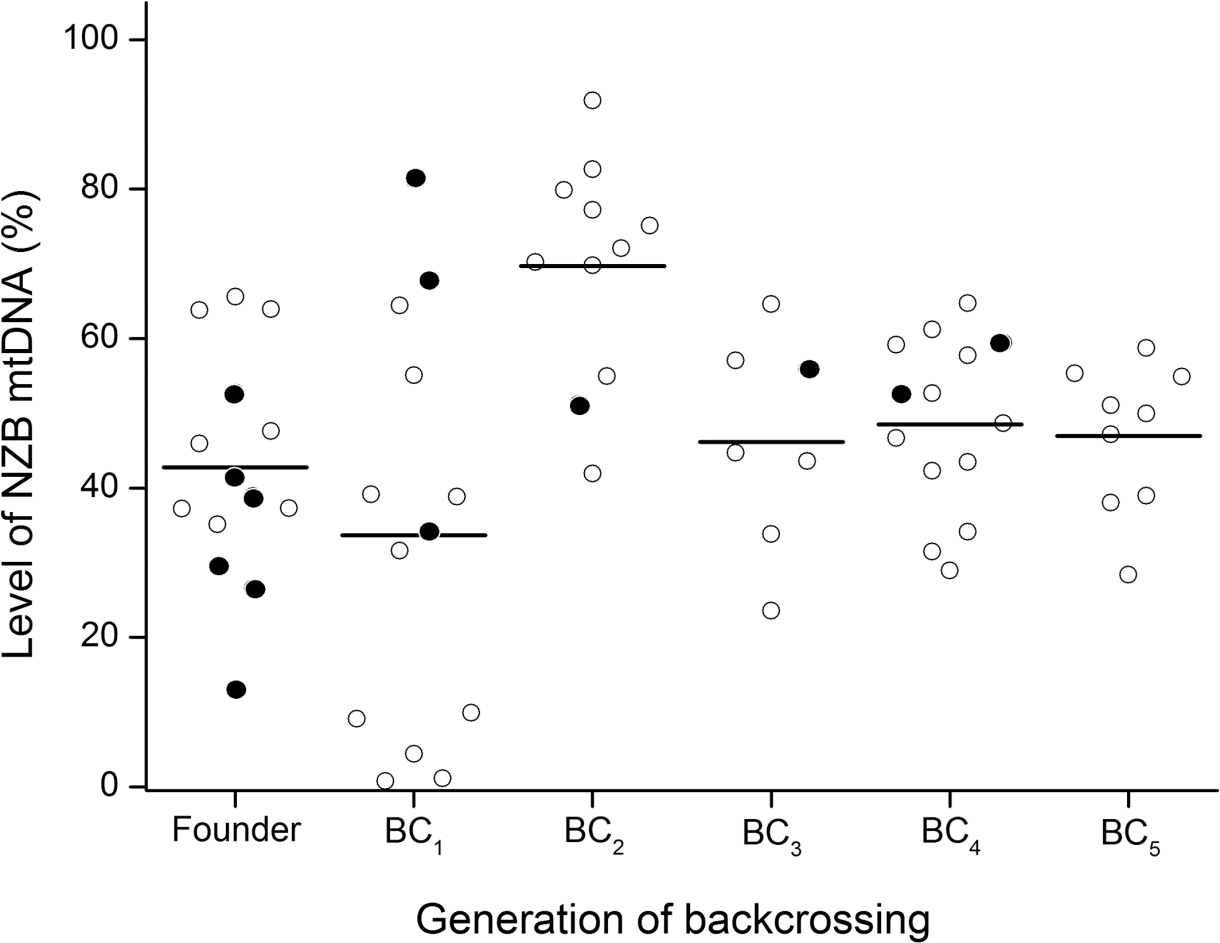
Level of NZB mtDNA across generations in a heteroplasmic mouse lineage produced by cytoplasmic transfer. Circles depict the level of NZB mtDNA in mice estimated by ARMS-qPCR from ear biopsies. Filled circles depict females that were backcrossed (BC) with B6 males to obtain the next generation progeny (BC_1_, BC_2_, BC_3_, BC_4_ and BC_5_). The level of NZB mtDNA was not determined for males, except in the founder lineage where males are depicted by empty circles. In the remaining generations (BC_1_, BC_2_, BC_3_, BC_4_ and BC_5_) empty circles depict females that were not mated. Bars represent the means.

**Fig 6:**
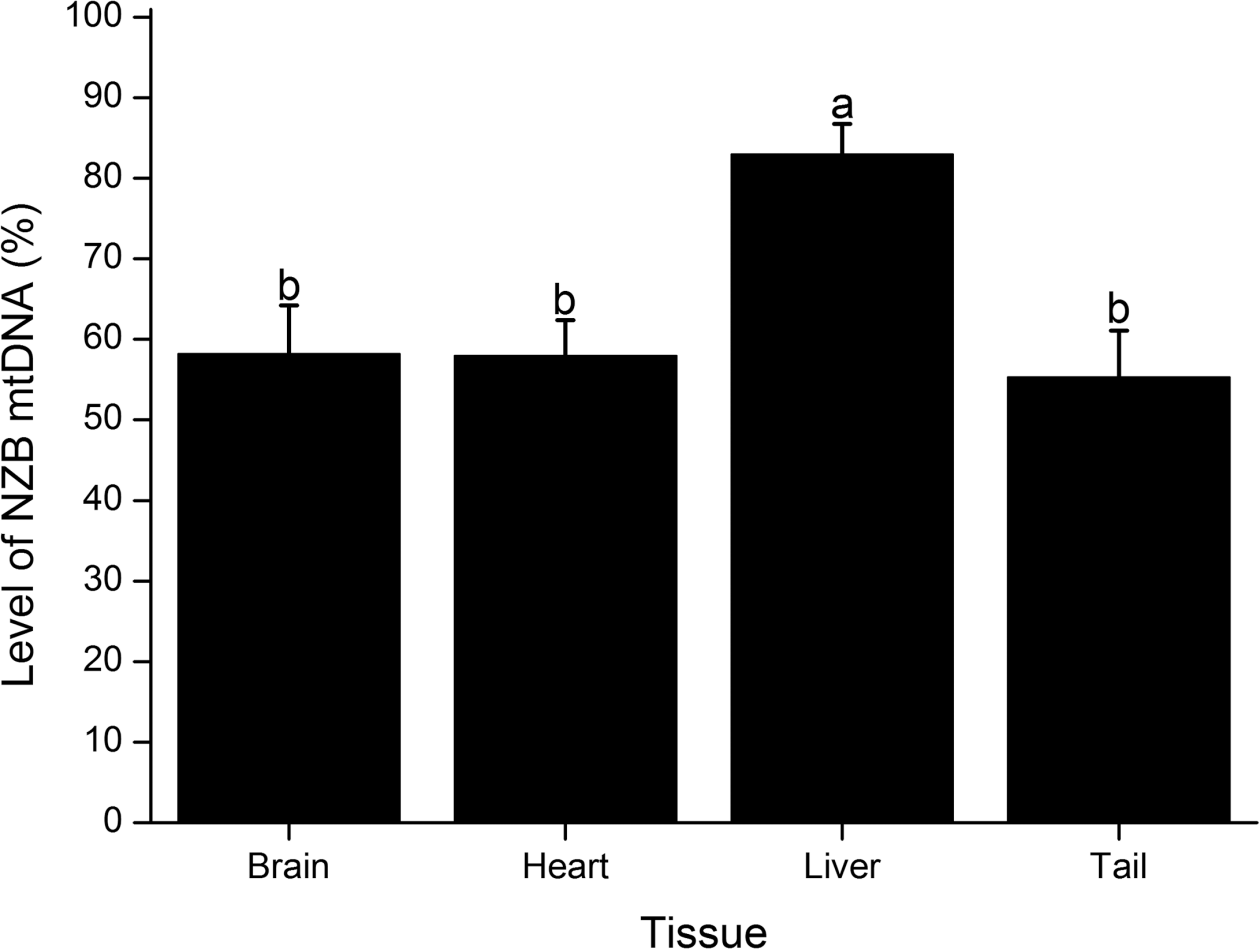
Comparison of the level of NZB mtDNA among tissues of heteroplasmic males. Bars depict the level of NZB mtDNA estimated by ARMS-qPCR from mice aged four months. Tissues analyzed included brain, heart, liver and tail. Values are reported as mean ± SEM. Bars with different letters denote a significant difference among tissues (P < 0.05).

In order to validate the ARMS-qPCR assay in somatic tissues, heteroplasmic zygotes from the centrifuged group were transferred to foster mothers that gave birth to 14 heteroplasmic pups (Fig 5). These founder mice contained on average 42.8% ± 3.92 (range, 13.0–65.6) of NZB mtDNA in tail, which did not differ (P > 0.05) from the level found in zygotes of the same experimental group (Fig 4). However, the level of NZB mtDNA in founders differed in tail (P < 0.05) between males (49.6% ± 4.72) and females (33.7% ± 5.45). Moreover, analysis of five heteroplasmic males aged four months provided evidence that the level of NZB mtDNA is variable among tissues (Fig 6). These males contained more NZB mtDNA (P = 0.004) in liver (83.0% ± 5.08) than in tail (55.3% ± 5.08), heart (58.0% ± 5.08) or brain (58.2% ± 5.08). With respect to founder females, these were mated with B6 males to obtain BC_1_ generation. Females from BC_1_ generation contained on average 33.7% ± 7.32 (range, 0.8–81.3) of NZB mtDNA, which did not differ from founder females (P > 0.05). To establish a heteroplasmic mouse lineage, three females from BC_1_ and their progeny were backcrossed for four generations with B6 males (Fig 5). The use of progenitors with intermediate levels of heteroplasmy resulted in females from BC_2_, BC_3_, BC_4_ and BC_5_ generations with 69.7% ± 4.45 (range, 41.9–91.9), 46.2% ± 5.00 (range, 23.6–64.6), 48.5% ± 3.18 (range, 29.0–64.7) and 47.0% ± 3.12 (range, 28.4–58.7) of NZB mtDNA, respectively. Three females from BC_4_ generation containing 31.5%, 46.7% and 59.2% of NZB mtDNA also had heteroplasmy measured in their oocytes, which contained, respectively, 22.4% ± 2.33 (range, 7.9–32.9), 41.9% ± 3.59 (range, 23.3–53.5) and 48.2% ± 7.28 (range, 21.7–65.4) of NZB mtDNA. Altogether, these results demonstrated the efficacy of the ARMS-qPCR assay for measurement of heteroplasmy, supporting its use in this mouse model.

### Analysis of mtDNA copy number in germline and somatic tissues

**Fig 7:**
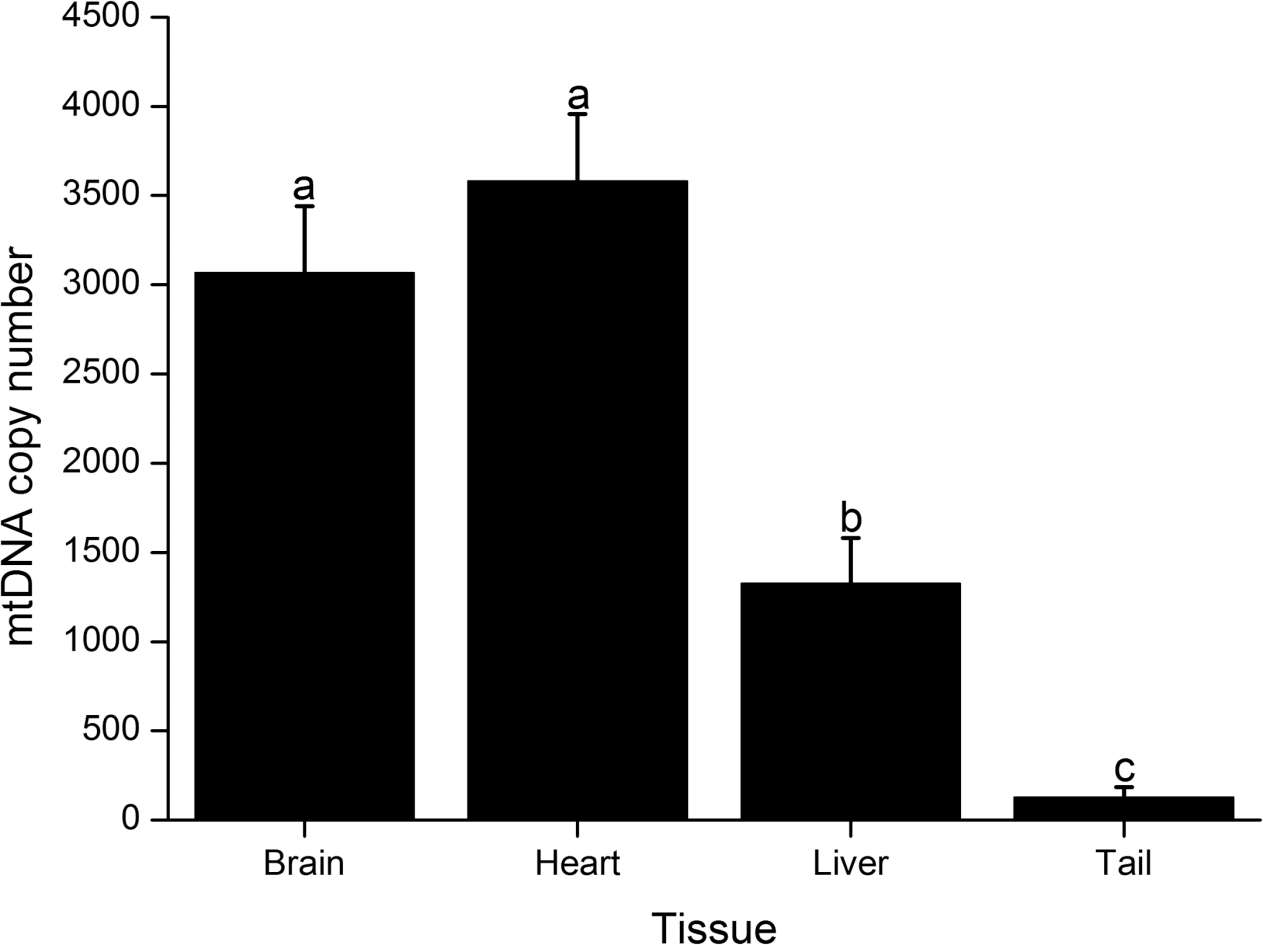
Comparison of mtDNA copy number among tissues of heteroplasmic males. Bars depict mtDNA copy number per cell from mice aged four months. Tissues analyzed included brain, heart, liver and tail. Values are reported as mean ± SEM. Bars with different letters denote a significant difference among tissues (P < 0.05).

As analysis of heteroplasmy is commonly accompanied by estimation of mtDNA copy number, we designed primers that non-discriminatively amplify mtDNA of NZB and B6 origin. These primers were used in conjunction with primers that amply a single copy nuclear gene (*Apob*) to measure mtDNA copy number in somatic tissues. However, we first compared amplification efficiency of these primer pairs (Fig 1) to demonstrate they were high (~100%) and similar (slope ≤ 0.1). Then, using the five heteroplasmic males described above we found that mtDNA copy number (Fig 7) is greater (P < 0.0001) in brain (3,066.4 ± 373.58; 2,335.0–4469.4) and heart (3,579.9 ± 375.55; 2470.5–4804.9) than in liver (1,326.8 ± 255.53; 660.9–2,236.8) and tail (126.8 ± 58.14; 24.5–225.8). Liver also contained more mtDNA copies than tail (P < 0.0001). This non-discriminative assay was also used to measure mtDNA in single oocytes. In this case, the single copy nuclear gene was not necessary as mtDNA copy number was estimated in relation to an external control. Analysis of heteroplasmic oocytes from three BC_4_ females indicated that these contained 344,738 ± 11,981 (range, 179,185–477,175) copies of mtDNA. In summary, these results support the use of our ARMS-qPCR assay for a rapid, sensitive and reliable quantification of mtDNA copy number and heteroplasmy in a mouse model with mtDNA of NZB and B6 origin.

## Discussion

Herein we report the use of ARMS-qPCR for quantification of heteroplasmy in a mouse model widely used in mitochondrial inheritance studies [5–13]. Our approach enables single-step quantification of heteroplasmy (i.e., quantification of either NZB or B6 mtDNA haplotypes), with sensitivity to detect as low as 0.1% of target mtDNA. Whereas quantification of heteroplasmy by PCR-RFLP takes several hours to be concluded (including PCR, labeling, restriction enzyme digestion, electrophoresis and analysis of signal intensity), analysis by ARMS-qPCR is completed within two hours as PCR, signal recording and analysis are all performed in a single step [13,19–23,25,26]. Moreover, although alternative labeling methods are available, radioactive labeling is still employed in last-cycle hot PCR-RFLP to avoid detection of heteroduplexes [18]. Therefore, the ARMS-qPCR approach represents a more powerful strategy for quantification of heteroplasmy.

Initially, as reported by others [13,20,22–24,29,38], we performed quantification of heteroplasmy by employing a discriminative (targeting B6 mtDNA) and a non-discriminative (targeting both NZB and B6 mtDNAs) assay. As a consequence, quantification accuracy was lower in samples with over 50% of NZB mtDNA (data not shown). The use of a discriminative assay targeting NZB mtDNA instead of the non-discriminative assay solved the problem, allowing measures of either NZB or B6 mtDNA haplotype from 0.1% to 99.9%, with high accuracy. Yet, a distinct SNP from the one targeted by the B6 assay (m.3932G>A) had to be utilized for the NZB assay (m.3599T>C). This was necessary as the presence of an adenine at position 3,599 of NZB mtDNA prevented the use of an ARMS assay with high specificity to amplify NZB mtDNA. On the other hand, the presence of a cytosine at position 3,932 of NZB mtDNA allowed the use of another ARMS assay capable of discriminating as little as 0.01% of NZB mtDNA, as in the case for the B6 assay. Moreover, the use of SYBR Green in qPCR provided a higher detection signal compared to the use of TaqMan probes (data not shown). Thus, considering the need to perform single cell quantification of heteroplasmy, the use of SYBR Green, besides being cost-effective, improved the assay sensitivity.

To validate our ARMS-qPCR approach, heteroplasmic zygotes were produced by cytoplasmic transfer using two methods that enable production of embryos with distinct levels of heteroplasmy [29]. According to our findings, embryos from the centrifuged group contained more NZB mtDNA than embryos from the non-centrifuged group. This result can be explained as centrifugation leads to formation of a mitochondrial-enriched cytoplasmic fraction [30–32] that can be removed from NZB embryos and transferred to B6 embryos from which the same cytoplasmic fraction was previously removed [29]. Hence, the use of centrifuged embryos enables generation of embryos with higher levels of hetereplasmy [29], which is in line with our present measurements. Transfer of zygotes from the centrifuged group to foster mothers enabled production of 14 mice, which contained on average 42.8% ± 3.92 of NZB mtDNA. As previously reported [5,8,10,13], the level of NZB mtDNA was expected to remain constant in tail, brain and heart while they increase with age in the liver. Analysis of NZB mtDNA haplotype from heteroplasmic males confirmed these previous findings, providing evidence of the reliability of our approach. Further evidence of this was provided by the finding that the level of NZB mtDNA was similar between heteroplasmic females and their oocytes or progenies, which is fully consistent with a random-drift model of mtDNA segregation [2,6,7,11].

At last, the number of mtDNA copies was measured in germline and somatic tissues using a non-discriminative assay. In oocytes, mtDNA copy number was estimated based on an external control, whereas in somatic tissues it was estimated in relation to a single copy nuclear gene. Estimation from both oocytes and somatic tissues provided results similar to those described in the literature [2,9,11,39–41]. As these assays for mtDNA copy number quantification were optimized for use under the same conditions as the assays for heteroplasmy quantification, both estimations can be performed in parallel providing a rapid and sensitive analysis.

In conclusion, based on the use of ARMS technology we report a qPCR assay for single-step quantification of heteroplasmy and mtDNA copy number from mice with mtDNA haplotype of NZB and B6 origin. This ARMS-qPCR assay was shown to be rapid, sensitive and accurate, enabling quantification of heteroplasmy from germline and somatic tissues to a limit of 0.1%. These findings are relevant as our ARMS-qPCR approach is fully compatible with similar heteroplasmic mouse models used previously to study mitochondrial inheritance in mammals [5–12].

## Supporting Information

S1 File ARMS-qPCR strategy. The scheme illustrates the rationale involving ARMS-qPCR technology.
(DOC)

S2 File Relationship between the input level of NZB gDNA and the level of NZB mtDNA estimated by ARMS-qPCR. Different proportions of gDNA from NZB mice (100%, 99.9%, 99.5%, 99%, 95%, 90%, 75%, 50%, 25%, 10%, 5%, 1%, 0.5%, 0.1% and 0%) were mixed with gDNA from B6 mice (input NZB%) to be analyzed by ARMS-qPCR (estimated NZB%).
(XLSX)

S3 File Level of NZB mtDNA in heteroplasmic mouse embryos produced by cytoplasmic transfer. Zygotes were centrifuged prior to cytoplasmic transfer to enable production of embryos with higher levels of NZB mtDNA. For comparison, cytoplasmic transfer was also performed without centrifugation. Embryos from both groups were evaluated with regards to the level of NZB mtDNA at the zygote and blastocyst stages by ARMS-qPCR.
(XLSX)

S4 File Comparison of the level of NZB mtDNA among tissues of heteroplasmic males. The level of NZB mtDNA was estimated by ARMS-qPCR from mice aged four months. Tissues analyzed included brain, heart, liver and tail.
(XLSX)

S5 File Level of NZB mtDNA across generations in a heteroplasmic mouse lineage produced by cytoplasmic transfer. The level of NZB mtDNA was estimated by ARMS-qPCR from ear biopsies. Females were backcrossed (BC) with B6 males for five generations to obtain the next generation progeny (BC_1_, BC_2_, BC_3_, BC_4_ and BC_5_). The level of NZB mtDNA was not determined for males, except in the founder lineage.
(XLSX)

S6 File Comparison of mtDNA copy number among tissues of heteroplasmic males. The number of mtDNA copies per cell was estimated from mice aged four months. Tissues analyzed included brain, heart, liver and tail.
(XLSX)
