# Supplementary material for: Real-Time PCR Quantification of Heteroplasmy in a Mouse Model with Mitochondrial DNA of C57BL/6 and NZB/BINJ Strains
Source: PLoS One. 2015 Aug 14;10(8):e0133650. doi: 10.1371/journal.pone.0133650 (PMC4537288; doi:10.1371/journal.pone.0133650)
Supplement: S1 File — The scheme illustrates the rationale involving ARMS-qPCR technology. (DOC) [file pone.0133650.s001.doc]

**ARMS-qPCR strategy**

Sequences below correspond to a fragment encompassing nucleotides 3,571 to 3,990 of B6 and NZB mtDNAs. Letters in red color depict polymorphic sites between B6 and NZB mtDNAs. Primers used to amplify B6 (ARMS2 and MT14) and NZB (ARMS22 and MT20) mtDNAs are depicted as yellow and green arrows, respectively. Discrimination between B6 and NZB mtDNAs is made by ARMS2 and ARMS22, respectively, which contain the terminal 3’-nucleotide specific to target haplotypes. In the presence of a non-target haplotype, the 3’ nucleotide will not match the target sequence preventing primer extension (illustrated by a red “X”). Moreover, arbitrary introduction of a mismatch to the penultimate nucleotide to the 3’ terminus of these primers (letter in red color) further increases their specificity.

***B6 mtDNA sequence***

**ARMS22**

**X**

TTATCCACGCTTCCGTTACGTC

||||||||||||||||||||

CTCGTAGAATAGGTGCGAAGGCAATGCTAGTTGAATATGTAGAAGATACTTTTTTGAAAGATGGGGATTG

GAGCATCTTATCCACGCTTCCGTTACGATCAACTTATACATCTTCTATGAAAAAACTTTCTACCCCTAAC

TGATCGTAATACATACACTGTATAAAGAAATGGTTAAAAATGTCGCCCTCATGGTGGTATGTATATCTTT

ACTAGCATTATGTATGTGACATATTTCTTTACCAATTTTTACAGCGGGAGTACCACCATACATATAGAAA

||||||||||||||||||||

AAAAATGTCGCCCTCACGGT

**MT20**

ATAGAGACTATTTTCTTAATGAAACTATCTCATTTAATATCTCCAAGTTCGGGAGAATAAAGATCCTGTT

TATGTCTGATAAAAGAATTACTTTGATAGAGTAAATTATAGAGGTTCAAGCCCTCTTATTTCTAGGACAA

**MT14**

CTCCGTGCTACCTAAACACCTTATC

|||||||||||||||||||||||||

ATCCTTAACTTGGATGTGAATTCTTAAGTTTTAAGAGGCACGATGGATTTGTGGAATAGGATTATCATTC

TAGGAATTGAACCTACACTTAAGAATTCAAAATTCTCCGTGCTACCTAAACACCTTATCCTAATAGTAAG

CAGTCGATTAATTCGATAGCCCGGGTATGGGGCTTTTGCAACCAAATTTAGGAAGGGCATGATTATTTAG

GTCAGCTAATTAAGCTATCGGGCCCATACCCCGAAAACGTTGGTTTAAATCCTTCCCGTACTAATAAATC

GATAGTGGGAACGGTAGTAGATGAAGTGTTAGAAGAATCCAGGACATTAGTGTTATAGGTCGTGGTTGGA

CTATCACCCTTGCCATCATCTACTTCACAATCTTCTTAGGTCCTGTAATCACAATATCCAGCACCAACCT

| |||||||||||||||||||||||||||

CTGTAGTAGATGAAGTGTTAGAAGAATCC

**ARMS2**

***NZB mtDNA sequence***

**ARMS22**

TTATCCACGCTTCCGTTACGTC

|||||||||||||||||||| |

CTCGTAGAATAGGTGCGAAGGCAATGCTGGTTGAATATGTAGAAGATACTTTTTTGAAAGATGGGGATTG

GAGCATCTTATCCACGCTTCCGTTACGACCAACTTATACATCTTCTATGAAAAAACTTTCTACCCCTAAC

TGATCGTAATACATACACTGTATAAAGAAATGGTTAAAAATGTCGCCCTCACGGTGGTATGTATATCTTT

ACTAGCATTATGTATGTGACATATTTCTTTACCAATTTTTACAGCGGGAGTGCCACCATACATATAGAAA

||||||||||||||||||||

AAAAATGTCGCCCTCACGGT

**MT20**

ATAGAGACTATTTTCTTAATGAAACTATCTCATTTAATATCTCCAAGTTCGGGAGAATAAAGATCCTGTT

TATGTCTGATAAAAGAATTACTTTGATAGAGTAAATTATAGAGGTTCAAGCCCTCTTATTTCTAGGACAA

**MT14**

CTCCGTGCTACCTAAACACCTTATC

|||||||||||||||||||||||||

ATCCTTAACTTGGATGTGAATTCTTAAGTTTTAAGAGGCACGATGGATTTGTGGAATAGGATTATCATTC

TAGGAATTGAACCTACACTTAAGAATTCAAAATTCTCCGTGCTACCTAAACACCTTATCCTAATAGTAAG

CAGTCGATTAATTCGATAGCCCGGGTATGGGGCTTTTGCAACCAAATTTAGGAAGGGCATGATTATTTAG

GTCAGCTAATTAAGCTATCGGGCCCATACCCCGAAAACGTTGGTTTAAATCCTTCCCGTACTAATAAATC

GATAGTGGGAATGGTAGTAGATGAAGTGTTAGAAGAATCCAGGACATTAGTGTTATAGGTCGTGGTTGGA

CTATCACCCTTACCATCATCTACTTCACAATCTTCTTAGGTCCTGTAATCACAATATCCAGCACCAACCT

**X**

|||||||||||||||||||||||||||

CTGTAGTAGATGAAGTGTTAGAAGAATCC

**ARMS2**
